# Exemestane Attenuates Hepatic Fibrosis in Rats by Inhibiting Activation of Hepatic Stellate Cells and Promoting the Secretion of Interleukin 10

**DOI:** 10.1155/2017/3072745

**Published:** 2017-12-10

**Authors:** Ya-Hui Wang, Rong-Kun Li, Ying Fu, Jun Li, Xiao-Mei Yang, Yan-Li Zhang, Lei Zhu, Qin Yang, Jian-Ren Gu, Xin Xing, Zhi-Gang Zhang

**Affiliations:** ^1^Shanghai Medical College of Fudan University, Shanghai 200032, China; ^2^State Key Laboratory of Oncogenes and Related Genes, Shanghai Cancer Institute, Ren Ji Hospital, School of Medicine, Shanghai Jiao Tong University, Shanghai 200240, China; ^3^Department of Oncology, Huai'an First People's Hospital, Nanjing Medical University, Huai'an 223300, China; ^4^Department of Obstetrics and Gynecology, Fengxian Hospital, Shanghai 201499, China

## Abstract

Exemestane (EXE) is an irreversible steroidal aromatase inhibitor mainly used as an adjuvant endocrine therapy for postmenopausal women suffering from breast cancer. Besides inhibiting aromatase activity, EXE has multiple biological functions, such as antiproliferation, anti-inflammatory, and antioxidant activities which are all involved in hepatic fibrosis. Therefore, we investigated the role of EXE during the progress of hepatic fibrosis. The effect of EXE on liver injury and fibrosis were assessed in two hepatic fibrosis rat models, which were induced by either carbon tetrachloride (CCl_4_) or bile duct ligation (BDL). The influence of EXE treatment on activation and proliferation of primary rat hepatic stellate cells (HSCs) was observed *in vitro*. The results showed that EXE attenuated the liver fibrosis by decreasing the collagen deposition and *α*-SMA expression *in vivo* and inhibited the activation and proliferation of primary rat HSCs *in vitro*. Additionally, EXE promoted the secretion of antifibrotic and anti-inflammatory cytokine IL-10 *in vivo* and in HSC-T6 culture media. In conclusion, our findings reveal a new function of EXE on hepatic fibrosis and prompted its latent application in liver fibrotic-related disease.

## 1. Introduction

Liver fibrosis is a wound-healing response to chronic injury. In this process, the activation and phenotype change of hepatic stellate cells (HSCs) are the key cellular events. HCSs change from vitamin A storing quiescent cells to proliferative and contractile myofibroblast-like cells producing extracellular matrix (ECM) [1]. Hepatic fibrosis tends to occur in men and postmenopausal women but rare in premenopausal women [2, 3]. Numerous evidences reveal that estrogen is the underlying factor of this phenomenon [4–6]. Accumulating studies have demonstrated that estrogen has protective effects for hepatic fibrosis and cirrhosis by inhibiting the activation and proliferation of hepatic stellate cells [7–9]. But unfortunately, except for its beneficial aspect, estrogen can also promote the growth of some malignant tumors. For example, targeting estrogen is one of the current effective therapeutic strategies for breast cancer.

Exemestane (EXE), an aromatase inhibitor, can decrease estrogen levels by blocking estrogen synthesis in the adipose tissue [10]. Presently, EXE is widely applied as an adjuvant endocrine therapy for estrogen-receptor- (ER-) positive postmenopausal women to prevent breast cancer and for advanced breast cancer after treatment failure with tamoxifen [11, 12]. In addition, to inhibit aromatase activity, EXE also had strong antiproliferative effect and can increase the occurrence of autophagy [11, 12]. The latest report revealed that EXE had anti-inflammatory and antioxidant activities, probably unrelated to aromatase inhibition [13]. Even more, Masri et al. suggested that EXE could induce estrogen receptor alpha activity [14]. Theoretically, EXE may have a promoting function for hepatic fibrosis through diminishing the estrogen levels. However, almost no patients who took EXE underwent hepatic fibrosis and cirrhosis. A recent study showed that aromatase inhibitor EXE decreased radiation-induced lung fibrosis, suggesting that it has a protective effect [15]. Until now, there is no study to investigate the exact role of EXE during the hepatic fibrosis process. In the field of experimental liver fibrosis research, the two most commonly used animal models are carbon tetrachloride (CCl_4_) and bile duct ligation- (BDL-) induced hepatic fibrosis models. CCl_4_ is the most widely used hepatotoxin in the study of liver fibrosis and cirrhosis in rodents. In many aspects, it mimics human chronic disease associated with toxic damage. Common bile duct ligation is well-known to cause cholestatic injury and periportal biliary fibrosis. To explore the role of EXE in liver fibrosis process, we applied EXE in CCl_4_ and BDL-induced hepatic fibrosis rat models and investigated its effects on collagen deposition and fibrotic marker expression. We further revealed its underlying mechanisms by exploring its roles in HSC activation, proliferation, and in the secretion of inflammatory factors.

## 2. Results

### 2.1. EXE Suppresses CCl_4_-Induced Rat Hepatic Fibrosis and Attenuates Hepatic Injury

**Figure 1:**
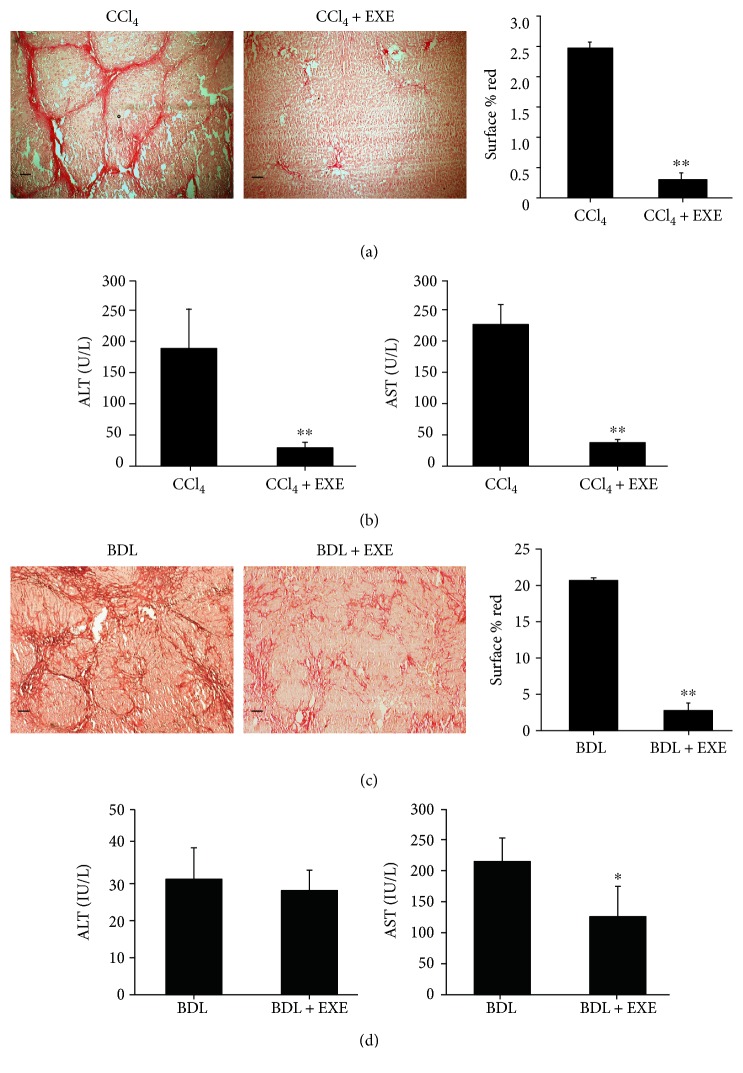
Effect of EXE administration on CCl_4_ and BDL-induced fibrosis in rats. (a) Representative images of liver tissues stained with Sirius Red are shown. Collagen deposition is indicated by the red strands and quantified using ImageJ software of six images. Scale bar 50 *μ*m. (b) Serum ALT and AST levels in the EXE-treated group and control group (10 rats/group). Data were expressed as means ± SD. (c) Collagen deposition was analyzed using Sirius Red staining and quantified six images using ImageJ software. Scale bar 50 *μ*m. (d) Serum ALT and AST levels in the EXE-treated group and control group (10 rats/group). Data were expressed as means ± SD. ^∗^*P* < 0.05, ^∗∗^*P* < 0.01.

To directly investigate the role of EXE on hepatic fibrosis, CCl_4_-induced rat hepatic fibrosis model was established. Rats were treated with CCl_4_ for 8 weeks with or without EXE treatment. After the rats were sacrificed, the liver fibrosis degree was assessed by Sirius Red staining. Quantification of Sirius Red-stained collagen areas in images of rat liver tissues clearly showed that EXE-treated rats had less collagen deposition than control rats (Figure 1(a)). To further evaluate the function of EXE for liver injury, we detected the serum ALT and AST levels, which are the serological markers for liver fibrosis and liver function. We found that rats treated with EXE had a notable reduction of serum ALT and AST levels compared to rats without EXE treatment (Figure 1(b)). These data indicate that EXE has a protective effect on hepatic fibrosis and liver injury.

### 2.2. EXE Ameliorates BDL-Induced Hepatic Fibrogenesis and Hepatic Injury

To validate our primary results, EXE was administered in BDL-induced rat hepatic fibrosis model. Consistent with the results of CCl_4_-induced model, EXE remarkably ameliorated the progression of BDL-induced liver fibrosis, as illustrated in the Sirius Red-stained liver specimens (Figure 1(c)). The serum AST levels were significantly decreased in the EXE-treated group. However, the serum ALT levels of the EXE-treated group had no significant alteration compared to the control group (Figure 1(d)). Our finding is further convinced by BDL-induced rat hepatic fibrosis model. Thus, EXE can significantly inhibit hepatic fibrosis *in vivo*.

### 2.3. EXE Inhibits the Expression of Profibrogenic Markers

**Figure 2:**
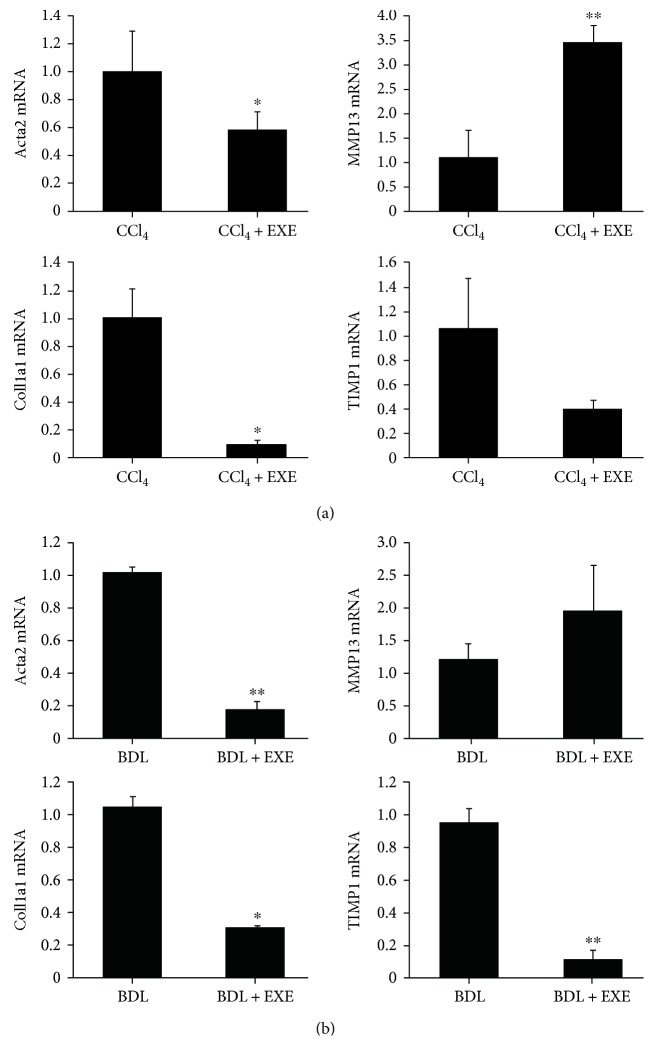
Influence of EXE on gene expression of profibrogenic markers in the liver. (a) Hepatic levels of Acta2, Coll1a1, MMP13, and TIMP1 mRNA in CCl_4_-induced fibrosis rats were determined using qPCR. (b) Hepatic levels of Acta2, Coll1a1, MMP13, and TIMP1 mRNA for BDL-induced fibrosis rats were determined using qPCR (10 rats/group). Data were expressed as means ± SD. ^∗^*P* < 0.05, ^∗∗^*P* < 0.01.

Meanwhile, the mRNA expression levels of Acta2, MMP13, Coll1a1, and TIMP1 were examined, which are all classical profibrogenic markers during liver fibrosis process [16, 17]. In CCl_4_-induced rat model, EXE significantly inhibited the expression levels of Acta2 and Coll1a1 and elevated the expression level of MMP13. Nevertheless, it had no obvious effect on TIMP1 expression (Figure 2(a)). Likewise, EXE significantly decreased the BDL-induced upregulation of Acta2, Coll1a1, and TIMP1, but had no obvious impact on MMP13 expression (Figure 2(b)).

### 2.4. EXE Inhibits HSC Activation and Proliferation *In Vivo*

**Figure 3:**
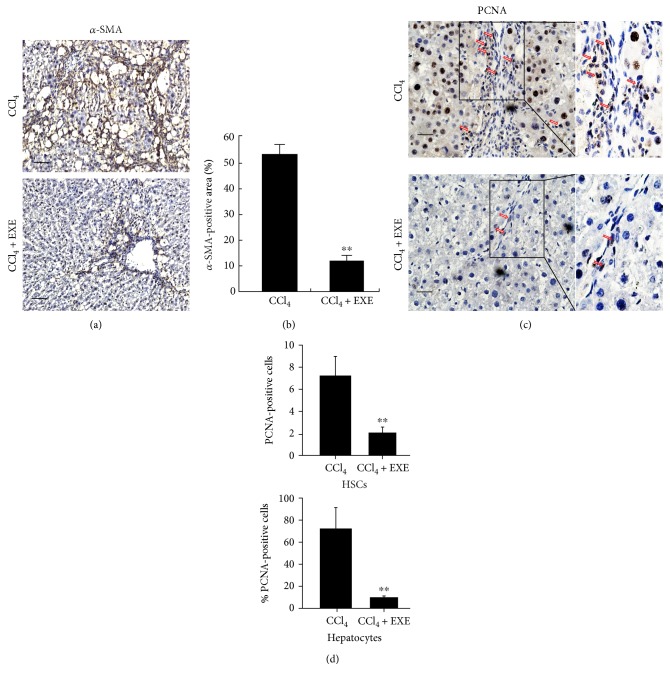
EXE inhibits the activation and proliferation of HSCs *in vivo*. (a) Immunohistochemical staining of *α*-SMA in liver tissues. Scale bar 50 *μ*m. (b) Quantification of *α*-SMA-positive area was shown as means ± SD of six images. ^∗∗^*P* < 0.01. (c) Immunohistochemical staining of PCNA in liver tissues. The red arrows indicate PCNA-positive nuclei in myofibroblasts. Scale bar 25 *μ*m. (d) Quantification of PCNA-positive HSCs and % PCNA-positive hepatocytes was shown as means ± SD of six images. ^∗∗^*P* < 0.01.

Hepatic fibrosis is accompanied with the activation of hepatic stellate cells (HSCs) [18]. The activated HSCs increase the fibrillar extracellular matrix production and decrease the degradation and remodeling. To further explore the mechanism of EXE suppressing hepatic fibrosis, we performed immunohistochemical staining for *α*-SMA, a marker of HSC activation, and PCNA, a marker of cell proliferation in the rat liver samples from both EXE-treated and control groups. The results showed that EXE significantly decreased the expression of *α*-SMA in the liver and the *α*-SMA-positive HSCs mainly located in the septa of fibrotic liver (Figures 3(a) and 3(b)). The images of PCNA immunohistochemical staining revealed that the number of the PCNA-positive HSCs and hepatocytes in the liver of the EXE-treated rats was significantly reduced than that in the control rats (Figures 3(c) and 3(d)). The reduction of PCNA-positive hepatocytes also suggested that the EXE could attenuate liver injury. HSC activation and proliferation are key and symbolic events during the process of hepatic fibrosis [19]. Thereby, the inhibitory of EXE for HSC activation and proliferation *in vivo* might be the probable mechanism of its mitigation impact on liver fibrosis.

### 2.5. EXE Restrains rHSC Activation and Proliferation *In Vitro*

**Figure 4:**
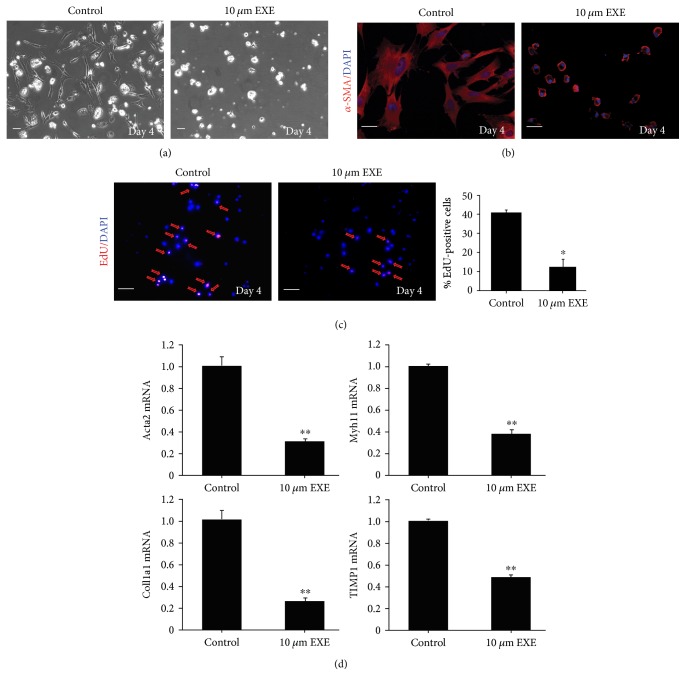
Effects of EXE on HSC activation and proliferation *in vitro*. (a) Bright field images of the control and EXE-treated HSCs at day 4 of culture. Scale bar 50 *μ*m. (b) EXE-treated and control cells were fixed at day 4 and immunostained with *α*-SMA antibodies. Scale bar 50 *μ*m. (c) At day 2, EXE-treated and control HSCs were exposed to EdU and fixed 2 days later and stained with Azide488 to visualize the DNA incorporated EdU. The percentage of EdU-positive cells was determined from three independent experiments. Scale bar 50 *μ*m. (d) mRNA levels of Acta2, Myh11, Coll1a1, and Timp1 in HSCs with or without EXE treatment were determined by qPCR (10 rats/group). Data were expressed as means ± SD. ^∗^*P* < 0.05, ^∗∗^*P* < 0.01.

To further investigate whether the inhibitory effect of EXE on fibrogenesis was due to the inhibition of HSC activation and proliferation, we incubated freshly isolated HSCs (rHSCs) from rats with or without EXE. Then, we investigated the effect of EXE on rHSC activation and proliferation. There is a clear discrepancy in morphological appearance of rHSCs treated with 10 *μ*M EXE for 4 days (Figure 4(a)); therefore, this concentration was performed for all treatments *in vitro*. rHSCs treated with 10 *μ*M EXE expressed significantly less *α*-SMA fibers and showed an inactivation phenotype in morphology compared with controls (Figure 4(b)). In accordance with the results obtained *in vivo*, EXE markedly decreased the proliferation of rHSCs by EdU staining (Figure 4(c)). Furthermore, the mRNA expression levels of Acta2, Coll1a1, TIMP1, and Myh11, which are classical markers for HSC activation, were significantly reduced in EXE-treated rHSCs (Figure 4(d)).

### 2.6. EXE Promotes the Secretion of Antifibrotic Cytokine IL-10

**Figure 5:**
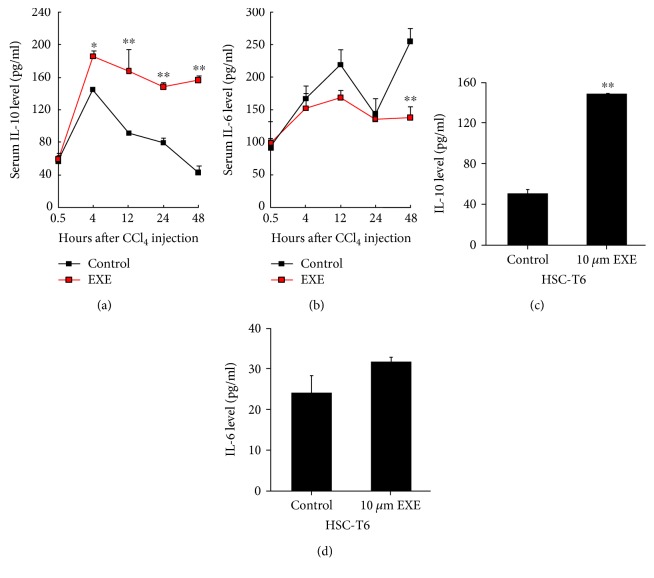
Effects of EXE on the secretion of IL-10 and IL-6 *in vivo* and *in vitro*. The serum levels of IL-10 (a) and IL-6 (b) were measured by ELISA after CCl_4_ injection with or without EXE treatment for 48-hour period (8 rats/group). The IL-10 (c) and IL-6 (d) levels in culture media of HSC-T6 cells treated with or without EXE for 48 h (*n* = 3). ^∗^*P* < 0.05, ^∗∗^*P* < 0.01.

Inflammation has a close relationship with chronic liver injury and hepatic fibrosis [20]. Numerous immunoregulatory cytokines are produced during the process of hepatic fibrosis and HSC activation. Among these cytokines, IL-10 has been proven to have a striking effect against liver injury and fibrosis [21, 22], and IL-6 is a key profibrotic factor [23]. To elucidate whether IL-10 and IL-6 are involved in the suppressing effect of EXE on hepatic fibrosis, we detected their serum levels in the control group and EXE-treated group. The results showed that the level of IL-10 was higher in the EXE group than in the control group (Figure 5(a)). In contrast, the EXE group showed less IL-6 than that of the control group (Figure 5(b)). Since IL-10 and IL-6 were partly secreted from activated HSCs in the impaired liver, we examined the concentration of IL-10 and IL-6 in HSC-T6 cells culture media before and after treatment with 10 *μ*M EXE. Likewise, EXE was also contributed to the secretion of IL-10, but not to the secretion of IL-6 *in vitro* (Figures 5(c) and 5(d)). Taken together, these data indicate that the antifibrotic effects of EXE are at least partially due to its anti-inflammatory activity.

## 3. Discussion

This study showed that treatment with EXE significantly attenuates CCl_4_ and BDL-induced hepatic fibrosis in male rats. EXE inhibited the activation and proliferation of HSCs *in vitro* and *in vivo* and promoted the secretion of antifibrotic cytokine IL-10. Our results explained why occurrence of hepatic fibrosis or cirrhosis is not elevated in breast cancer patients who were administered with EXE, though it suppresses estrogen, and revealed that EXE might be a potential drug for treatment of hepatic fibrosis or cirrhosis.

Hepatic fibrosis or cirrhosis is a common clinical situation that has various aetiologies and serious impairment for patients, but satisfactory therapies are still lacking. Sustained and exaggerated inflammation and hepatic stellate cell activation are the core processes during hepatic fibrosis initiation and progression [24]. EXE is a widely used drug for estrogen receptor-positive postmenopausal breast cancer patients. Our findings displayed its inhibitory effect in CCl_4_ and BDL-induced hepatic fibrosis rat models by suppressing HSC activation and proliferation. Thus, it might be very interesting to make further clinical analysis of EXE on hepatic fibrosis and cirrhosis prevention and therapy.

The roles of estrogen in hepatic fibrosis and cirrhosis are still controversial, but dominating evidences tend to support the idea that estrogen plays protective effects against hepatic fibrosis [5]. As expected, EXE should promote hepatic fibrosis as it is an aromatase inhibitor and can decrease the estrogen levels. Surprisingly, our results showed that EXE significantly suppressed hepatic fibrosis *in vivo*. And *in vitro*, our assay displayed that EXE also inhibited HSC activation. This suggested that the antifibrotic effect of EXE is not related to aromatase inhibition, but due to its inhibitory effects on HSC activation and proliferation.

Liver inflammation is the hallmark of early-stage liver fibrosis, ultimately resulting in HSC activation and ECM deposition. In the numerous immunoregulatory cytokines, IL-10 is a potent anti-inflammatory cytokine. It can repress proinflammatory responses and limit unnecessary tissue disruptions caused by inflammation. By ELISA analysis, we found that IL-10 level was significantly elevated in the serum of EXE-treated rats. Therefore, it revealed that modulating anti-inflammatory cytokines might partly be the mechanisms of antifibrotic effects of EXE. However, inflammatory factors are mainly secreted by local inflammatory cells in the liver, such as Kupffer cells and lymphocytes. Hence, further researches about EXE on inflammatory cell infiltration and anti-inflammatory cytokine secretion are under investigation.

In summary, our *in vitro* and *in vivo* findings demonstrated that treatment with EXE attenuates the process of hepatic fibrosis by inhibiting the activation of HSCs and upregulating the secretion of IL-10. To our knowledge, this study is the first to show the antifibrotic effects of EXE. As a widely applied medicine for breast cancer, EXE has shown no significant side-effects on renal or liver functions [25]. Therefore, EXE might be useful as an antifibrotic agent in chronic liver diseases. There are many common processes existing in different organ fibrotic diseases, including interstitial cell activation and inflammatory factor dysregulation. Thereby, EXE might also have extensive potential application in other different organ fibrotic diseases, such as renal fibrosis, pulmonary fibrosis, scleroderma, and so on.

## 4. Methods

### 4.1. Cell Culture and Reagents

HSC-T6 cells, an immortalized rat HSC cell line, were obtained from the cell bank of the Chinese Academy of Sciences (Shanghai, China) and cultured in Dulbecco's modified Eagle's medium (DMEM, Gibco, USA) supplemented with 10% fetal calf serum (HyClone, Australia), 100 U/ml penicillin, and 100 *μ*g/ml streptomycin. EXE was obtained from Sigma (St. Louis, USA).

### 4.2. Animal Experiments

The hepatic fibrosis models of male Sprague-Dawley (SD) rats were induced by CCl_4_ and BDL, respectively. For CCl_4_-induced hepatic fibrosis model, 20 SD rats about 200 g were given intraperitoneal injections of 1 ml CCl_4_/kg body weight diluted 1 : 1 in olive oil twice weekly for 8 weeks. One week after the first injection of CCl_4_, SD rats were randomly divided into two groups: CCl_4_ group and EXE group (EXE + CCl_4_) (*n* = 10 in each group), and started to treat with EXE. For BDL liver fibrosis model, 20 male SD rats were also divided into two groups one week after the rats underwent BDL (*n* = 10 in each group) and started to receive EXE or vehicle treatment. EXE was suspended in normal saline solution and administered twice a week by intraperitoneal injection at 4 mg/kg body weight. Rats were sacrificed 48 hours after the last EXE injection. All rat livers were fixed in formaldehyde or immediately frozen in liquid nitrogen. Rats were manipulated and housed according to protocols approved by the East China Normal University Animal Care Commission. All animals received humane care according to the criteria outlined in the “Guide for the Care and Use of Laboratory Animals” prepared by the National Academy of Sciences and published by the National Institutes of Health.

### 4.3. Isolation and Culturing of Rat HSCs

Male SD rats (250 to 300 g) were used to isolate and purify HSCs. Rat HSC isolation method was a modification of the previously described method [26]. Rat HSCs were isolated using a two-step collagenase/pronase E perfusion of rat livers, followed by 18% Nycodenz two-layer discontinuous density-gradient centrifugation. After isolation, cells were suspended in DMEM with 10% fetal calf serum, 100 U/ml penicillin, and 100 *μ*g/ml streptomycin, plated on plastic dishes (Corning, USA), and cultured at 37°C in a humidified atmosphere with 5% CO_2_ and 95% air. Culture medium was renewed 24 hours after plating.

### 4.4. Measurement of Liver Enzymes and Cytokines

Rat blood was collected in Eppendorf tubes and standing in 4°C overnight. The serums were separated by centrifugation at 3000 r/min for 20 minutes and stored at −80°C. Serum ALT and AST activities were detected with ALT and AST kits (Shen Suo You Fu Co. Ltd., Shanghai, China) by an automated analyzer. To study the effects of EXE on anti-inflammatory cytokines, such as IL-10 and IL-6, rat blood was collected from the rat tails at 0.5 h, 4 h, 12 h, 24 h, and 48 h after the last injection of CCl_4_. At each time point, there were IL-10 and IL-6 levels that were quantitated with ELISA kits (eBioscience, USA) according to manufacturer's instructions. For *in vitro* experiments, after 48 hours of treatment with EXE, the IL-10 and IL-6 levels in culture media of HSC-T6 cells were detected.

### 4.5. Quantitative RT-PCR

Total RNA from liver tissues and cells was extracted using TRIzol (Takara, China) and reverse-transcribed using the PrimeScript™ RT reagent kit (Takara, China) according to the manufacturers' instructions. Quantitative PCR was carried out on the ABI 7300 sequence detector (Applied Biosystems, Rotkreuz, Switzerland). The primer sequences used are summarized in Table 1. Gene expression values were calculated based on −△Ct method and normalized to expression of GAPDH. Results were calculated as 2^−△△Ct^ and express the *x*-fold increase of gene expression compared to control rats or cells.

### 4.6. Cell Proliferation Assay

Cell proliferation was investigated by measuring active DNA synthesis with the Cell-Light™ EdU Apollo®567 cell tracking kit (RiboBio, Guangzhou, China). Isolated primary HSCs were seeded in 48-well plates containing round coverslips at density of 5000 cells/well with the presence or absence of 10 *μ*M EXE. After 48 hours, EdU labeling was initiated. Another 48 hours later (day 4), cells were formalin fixed and visualization of the EdU incorporation was obtained according to the manufacturer's instructions.

### 4.7. Sirius Red Staining and Immunohistochemistry

Liver specimens were fixed in 10% formalin. To detect collagen fibers, paraffin-embedded liver sections were stained in 0.1% Sirius Red F3BA in a saturated picric acid solution. Randomly selected five fields from each section were photographed and analyzed. Red staining areas were quantified using NIH ImageJ software (http://rsb.info.nih.gov/ij/) and expressed as a percentage of total analyzed areas. For immunohistochemical staining, all tissue samples were fixed in phosphate-buffered neutral formalin, embedded in paraffin, and then cut into 5 *μ*m thick sections. Tissue sections were deparaffinized and rehydrated and then incubated with 0.3% hydrogen peroxide/phosphate-buffered saline for 30 minutes and blocked with 10% BSA. Slides were first incubated using the mouse-anti-*α*-smooth muscle actin (*α*-SMA) antibody (clone 1A4; Sigma-Aldrich, USA) and a rabbit-anti-proliferating cell nuclear antigen (PCNA) antibody (ab29, Abcam, USA), respectively, at 4°C overnight with optimal dilution, labeled by HRP second antibody (A-11059 and A-21245, Thermo Scientific, USA) at room temperature for 1 hour, incubated with DAB substrate liquid (Thermo Scientific), and counterstained with hematoxylin. All sections were observed and photographed with a microscope. *α*-SMA-positive staining areas were quantified using ImageJ software and presented as a percentage of total analyzed areas. PCNA-positive and negative cells were counted at ×400 magnification. All reactive cells were counted as positive regardless of the intensity of staining. For HSCs, PCNA-positive cells were counted and expressed. For hepatocytes, in each picture, all cells were counted and the percentage of positive hepatocytes was determined.

### 4.8. Statistical Analysis

Data are expressed as means ± standard deviation of at least three independent experiments unless indicated otherwise. Groups are compared using a two-tailed Student *t*-test. *P* < 0.05 is considered statistically significant.

## Figures and Tables

**Table 1:** Oligonucleotide sequences of primers used in real-time PCR.

Gene	Sequences (5′ → 3′)
MMP13	F:GGTTGAGCCTGAACTGTTTTTGA
	R:CTCGTATGCAGCATCCACATG
ColI1a1	F:CAGGCTGGTGTGATGGGATT
	R:CCAAGGTCTCCAGGAACACC
TIMP1	F:GACCACCTTATACCAGCGTT
	R:GTCACTCTCCAGTTTGCAAG
Acta2	F:GGACGTACAACTGGTATTGTGC
	R:CGGCAGTAGTCACGAAGGAAT
Myh11	F:TGAGAGGAAGAAGATGGCTCA
	R: TGTAGTTTCTGTCTGGCAGCTT
GAPDH	F:GCTGAGTATGTCGTGGAGTCT
	R:GGTTCACACCCATCACAAACA
